# Surgical treatment of a rare case of hepatocellular carcinoma with right atrial metastasis: A case report

**DOI:** 10.1097/MD.0000000000021630

**Published:** 2020-08-07

**Authors:** Wei Qiu, Chuanlei Wang, Ruoyan Zhang, Feng Wei, Xiaoju Shi, Xiaodong Sun, Dashi Ma, Guoyue Lv, Guangyi Wang

**Affiliations:** Department of Hepatobiliary and Pancreatic Surgery, The First Hospital of Jilin University, Changchun, Jilin, China.

**Keywords:** hepatocellular carcinoma, Inferior vena cava tumor thrombus, intracavitary metastasis, alpha-fetoprotein

## Abstract

**Rationale:**

Hepatocellular carcinoma (HCC) with intracavitary metastasis extending to the heart, also known as inferior vena cava (IVC) tumor thrombus, is an extremely rare late-stage disease with no effective treatment. In fact, the median survival is reportedly less than 2 months; thus, there is an urgent need for better treatment.

**Patient concerns:**

In this study, a 48-year-old patient was admitted to our hospital to seek medical treatment for advanced primary HCC with right atrial metastasis.

**Diagnosis:**

The patient was diagnosed as primary HCC with a large mass in the right lobe of the liver and intracavitary metastasis to the right atrium.

**Interventions:**

A new surgical treatment of right hemihepatectomy, complete resection of the involved IVC and the right atrium thrombus, plus reconstruction of the resected IVC using autologous pericardial tube graft were undertaken and successfully performed.

**Outcomes:**

The patient recovered rapidly, and 14 days after the surgical procedures, he was discharged from the hospital. Notably, serum levels of alpha-fetoprotein dropped to normal range and no clinical signs of recurrence were observed during follow-up.

**Lessons:**

This report highlights an unusual case of right atrial metastasis from HCC. The surgical treatment appeared to be suitable and effective, together with postoperative administration of lenvatinib, a tyrosine kinase multitarget inhibitor selected by performing whole-exome sequencing. These therapies have offered favorable clinical outcomes such as prevention of recurrence and prolongation of patient survival. In addition, clinicians may benefit from our experience for their future treatment of patients with similar clinical conditions.

## 1 Introduction

Extrahepatic metastasis of hepatocellular carcinoma (HCC) into intracavitary cardiac extension rarely occurs in patients with HCC, and tumor thrombus (TT) extending through the inferior vena cava (IVC) into the heart chambers (e.g., right atrium) is even more rare.^[1–4]^ Because the disease is associated with a high risk of sudden death, an effective and timely treatment is needed.

It is generally accepted that HCC patients with IVS tumor thrombus (IVCTT) are not suitable candidates for surgical treatment, and alternatively, transarterial-chemoembolization (TACE) as a palliative treatment or systemic chemotherapy is widely performed in patients.^[5,6]^ However, current treatment approaches have been far from satisfactory, and the prognosis of these patients is extremely poor with a median survival less than 2 months, which is shorter than that of HCC patients with portal vein TT. Thus, there is an urgent need for an appropriate and better treatment.

In this study, we present an extremely rare case of HCC with intracavitary metastasis to the right atrium, in which a new surgical treatment of right hemihepatectomy, resection of the involved IVC and the right atrium thrombus, plus reconstruction of the resected IVC were performed. We also discussed the suitability, feasibility, and effectiveness of the surgical procedures to potentially be used in similar conditions.

## 2 Case report

### 2.1 Patient description

**Figure 1:**
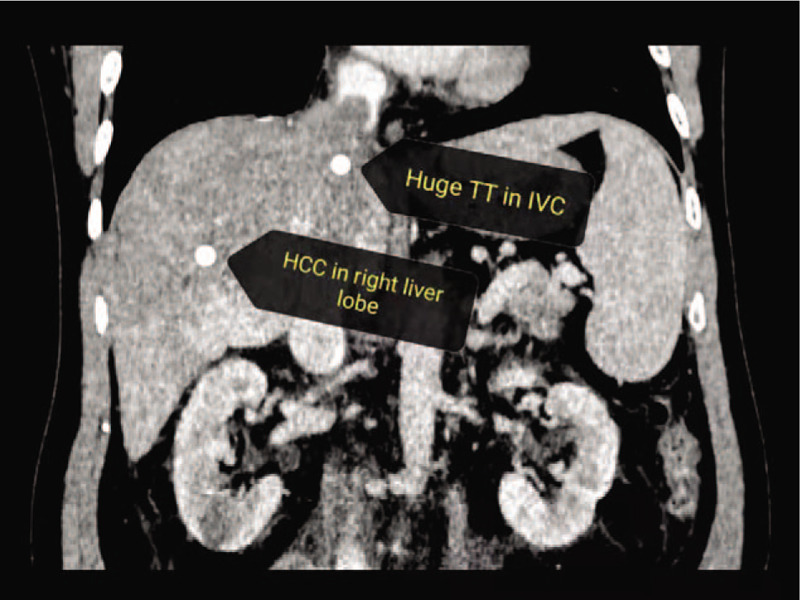
Computed tomography imaging of primary hepatocellular carcinoma and metastatic cardiac mass. HCC = hepatocellular carcinoma, TT = tumor thrombus, IVC = inferior vena cava.

**Figure 2:**
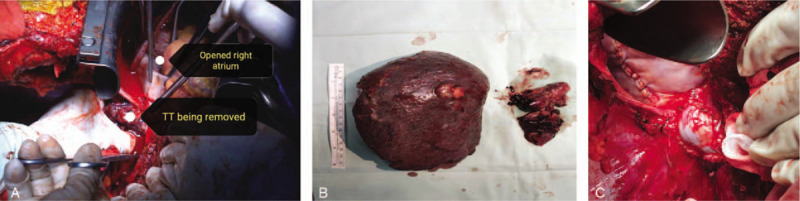
Intraoperational observations. A, Right atrial metastasis of the primary hepatocellular carcinoma. B, Gross specimen of resected cardiac mass. C, Reconstruction of resected vascular segment of inferior vena cava with autologous pericardial tube graft.

A 48-year-old man, who was diagnosed with advanced primary HCC with right atrial metastasis and sought medical treatment, was admitted to our hospital. A computed tomography scan revealed a large mass (approximately 7.6 × 7.6 cm in size) in the right lobe of the liver and intracavitary metastasis to the right atrium, an extremely rare secondary cardiac malignancy of primary HCC metastasis (Fig. 1). According to the classification of IVCTT as previously described,^[7]^ the present case was intra-cardiac type III IVCTT with a cardiac TT above the diaphragm that extended to the right atrium of the heart. The patient underwent surgical treatment on September 25, 2018, during which time right atrial metastasis in the heart was actually observed, extending from the right liver via the IVC to the right atrium (Fig. 2A); the gross specimen of resected cardiac mass is shown in Figure 2B.

### 2.2 Treatment and clinical outcome

After full assessment, surgical treatment, consisting of hepatectomy, resection of the involved IVC and right atrium thrombus, and reconstruction of the resected IVC, was performed in this patient. After blocking the right Glisson pedicle and marking the ischemic line at the junction of the left and right half liver, the liver was split along the line under the blocking of the first hepatic hilar and subhepatic IVC. The right Glisson's pedicle was then dissected. Subsequently, the chest was opened, the mediastinum and the diaphragm muscles around the hepatic IVC were cut, the pericardium was opened, allowing fully exposure of the heart, and cardiopulmonary bypass (CPB) was performed in the patient. In the following operative procedures, the right atrium was cut, the right half liver was removed at the root of the right hepatic vein; a longitudinal 8-cm long incision was made on the surface of the superior hepatic IVC, followed by complete removal of the IVCTT and secondary thrombosis. The right atrium incision was closed and the resected IVC was then reconstructed in which autologous pericardial tube graft was selected and used for suitability mainly due to its low thrombogenicity, low risk of narrowing, and contracture. The patient agreed to the treatment plan, and the operational procedures were performed by a surgical team of hepatic and cardiothoracic surgeons under the condition of beating heart CPB (Fig. 2C). The entire operation lasted nearly 9 hours with no major procedure-related complications.

**Figure 3:**
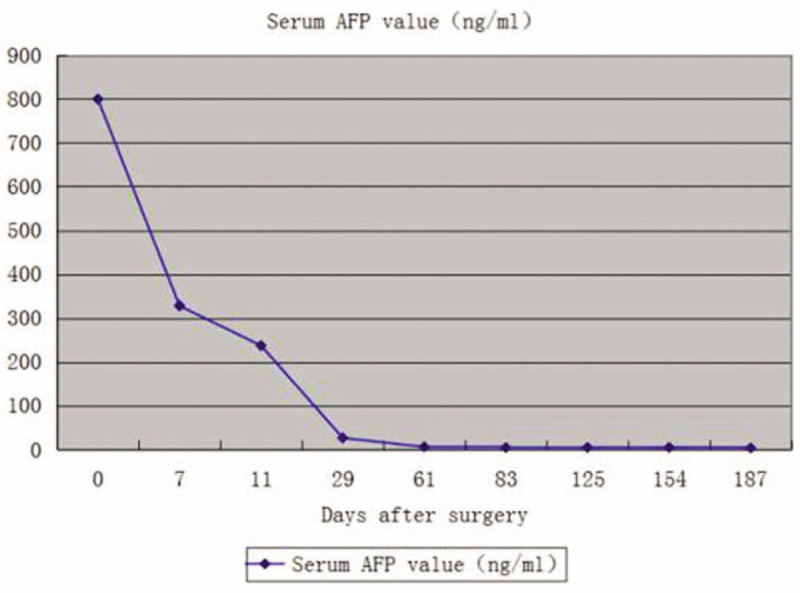
Follow-up examination of serum alpha-fetoprotein levels in the patient. AFP = alpha-fetoprotein.

Upon completion of surgical treatment, whole exome sequencing was performed to identify specific targeted cancer therapies for the patient. Levotinib, a tyrosine kinase multitarget inhibitor, was orally administered 12 mg once per day. Fourteen days after surgery, the patient was discharged from the hospital. Notably, the follow-up examination of serum alpha-fetoprotein (AFP) levels showed that they were within normal range (Fig. 3). Fortunately, the patient did not show any clinical signs or symptoms of recurrence during follow-up, and he is currently cancer free nearly 10 months after surgical treatment combined with targeted cancer therapy.

## 3 Discussion

Portal vein TT metastasized from HCC occur in a proportion of HCC patients, while HCC metastasis to the heart along the IVC has been uncommon.^[1–3]^ To date, there has been limited understanding of the clinical features and effective treatments for this disease. In this case report, we illustrated an extremely rare case of right atrial metastasis from HCC. Furthermore, the feasibility and suitability of the surgical procedures, in particular, complete resection of the involved IVC and reconstruction of the resected IVC using autologous pericardial tube graft was demonstrated in this patient. In general, these surgical procedures should be considered to treat similar patients, with the following main indications: liver cancer with IVCTT, no evidence of other extrahepatic metastasis, normal liver function, surgical operation expected to completely remove liver cancer and TT in IVC, patients with a strong desire for surgical treatment, and cooperation with comprehensive tumor treatments, including targeted therapy and immunotherapy after surgery.

It has been widely accepted that patients with advanced HCC with IVCTT are not suitable candidates for surgical treatment, whereas systemic chemotherapies and TACE as palliative care to improve quality of life are commonly used to manage these patients.^[5,6]^ Until now, no consensus regarding treatment strategies has been reached, and the overall survival of patients treated differently has shown different results.^[4,8–13]^ Mass excision of cardiac metastasis from HCC has offered favorable clinical outcomes, including a reduction of risk or incidence of sudden cardiac death and pulmonary embolism, suggesting the benefit of surgical removal of IVCTT if obstruction of outflow or inflow tracts is present. However, recurrence has occurred in these patients. In our patient, a combination of surgical procedures was undertaken after previous studies were reviewed.^[14–16]^ The following experiences regarding the complete resection of IVCTT and involved vascular segment of IVC, and reconstruction of the resected IVC in the present case study may merit attention within the field. Under full preoperative assessment of HCC with IVCTT, complete resection of IVC wall invasion by TT or involved vascular segment is highly recommended to reduce the possibility of TT falling off the IVC wall and to avoid recurrence, because we are inclined to believe that, in HCC patients with IVCTT, IVCTT is derived from invasion of HCC into IVC through hepatic veins or short-length hepatic veins. In replacement of the resected vascular segment, autologous pericardial tube graft is suitable for reconstruction and is highly recommended in similar cases. In addition to autologous pericardial tube graft, the iliac vessels from the donors of donation after circulatory death (DCD) could also be used for reconstruction of the resected vascular segment. In fact, in our liver transplantation center, we routinely reserve iliac vessels from DCD donors, which may facilitate the surgical treatment of such patients. During the surgical procedures, hepatectomy should be performed before IVCTT is resected, as we and other clinicians previously suggested.^[8,12]^ This is particularly important to reduce bleeding and other potential complications during the surgical treatment of IVCTT, in particular, intracardiac type III IVCTT, in which surgical procedures are conducted under the condition of beating heart CPB. Given the complex surgical procedures, a surgical team consisting of hepatic and cardiothoracic surgeons is highly recommended to ensure that the operations are successfully conducted. Postoperative monitoring of serum AFP may help with the evaluation of clinical outcomes of treatment. Actually, the levels of AFP rapidly declined following the surgical treatment with both tumor markers falling within normal range, which was in agreement with the clinical observations of no recurrence in our patient. Previous studies have reported that the median overall survival of patients with surgery was 12 to 19 months, whereas that of patients with other treatments (e.g., TACE, radiation therapy, chemotherapy) was 4.2 to 10 months.^[11,17–19]^ These results suggested that the patients who underwent surgical treatment had greater overall survival compared with those patients with other treatments.

In conclusion, we have reported an extremely case with HCC metastasis to the right atrium, and surgical treatment was effective and offered favorable clinical outcomes. As such, surgeons may benefit from our experience of suitable surgical treatment in combination with postoperative targeted cancer therapy for the future treatment of similar patients.

## Author contributions

WQ and GYW were major contributors in writing the original manuscript. CLW and RYZ contributed to data collection and analysis. FW, XJS, XDS, GYL, and DSM were major participants of the operation, and were responsible for the surgical treatment and methodology. All authors read and approved the final manuscript.
